# Viral Bad News Sent by EVAIL

**DOI:** 10.3390/v13061168

**Published:** 2021-06-18

**Authors:** Matthias Clauss, Sarvesh Chelvanambi, Christine Cook, Rabab ElMergawy, Navneet Dhillon

**Affiliations:** 1 IU School of Medicine, Pulmonary, Critical Care, Sleep and Occupational Medicine, Indianapolis, IN 46202, USA; rgelmerg@iu.edu; 2 Brigham and Women’s Hospital, Department of Medicine, Boston, MA 02115, USA; schelvanambi@bwh.harvard.edu; 3 Pulmonary and Critical Care Medicine, University of Kansas Medical Center, Kansas City, KS 66160, USA; ccook3@kumc.edu (C.C.); NDHILLON@kumc.edu (N.D.)

**Keywords:** extracellular vesicles, viral infection, HIV, SARS-CoV-2, Nef, antiretroviral therapy, cardiovascular disease

## Abstract

This article reviews the current knowledge on how viruses may utilize Extracellular Vesicle Assisted Inflammatory Load (EVAIL) to exert pathologic activities. Viruses are classically considered to exert their pathologic actions through acute or chronic infection followed by the host response. This host response causes the release of cytokines leading to vascular endothelial cell dysfunction and cardiovascular complications. However, viruses may employ an alternative pathway to soluble cytokine-induced pathologies—by initiating the release of extracellular vesicles (EVs), including exosomes. The best-understood example of this alternative pathway is human immunodeficiency virus (HIV)-elicited EVs and their propensity to harm vascular endothelial cells. Specifically, an HIV-encoded accessory protein called the “negative factor” (Nef) was demonstrated in EVs from the body fluids of HIV patients on successful combined antiretroviral therapy (ART); it was also demonstrated to be sufficient in inducing endothelial and cardiovascular dysfunction. This review will highlight HIV-Nef as an example of how HIV can produce EVs loaded with proinflammatory cargo to disseminate cardiovascular pathologies. It will further discuss whether EV production can explain SARS-CoV-2-mediated pulmonary and cardiovascular pathologies.

## 1. Introduction

**Figure 1:**
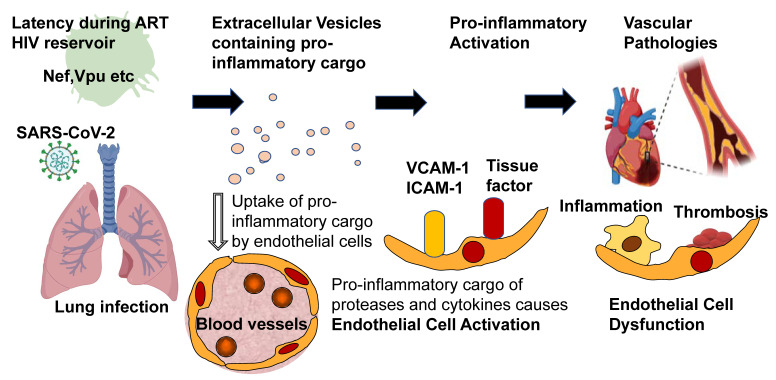
Proposed role of EVs in HIV-associated CVD and SARS-CoV-2 caused pathologies.

For many years, acute and chronic viral infections have been associated with the development of cardiovascular disease (CVD). For example, infectious encounters with cytomegalovirus (CMV) correlate with a 22% increased risk for future development of cardiovascular disease [1]. However, the contribution and the biology of viruses towards CVD are incompletely understood. What is extraordinarily well studied is infection with Human Immunodeficiency Virus-1 (HIV) and how it may affect cardiovascular health. Notably, after the introduction of antiretroviral therapy (ART), cardiovascular disease (CVD) became a leading cause of death in the HIV-positive population [2]. Because the HIV biology concerning cardiovascular and pulmonary diseases is so well understood, it may serve as a blueprint for studying other viruses with known impacts on CVD. This review focuses specifically on the role of extracellular vesicles (EVs) in HIV infection and their possible CVD contribution. Finally, using the example of the pandemic SARS-CoV-2 virus, it tries to apply what we have learned about HIV-induced EV formation to understand SARS-CoV-2 virus-induced CVD (see Figure 1).

## 2. HIV Mystery: Heightened CVD Risk in HIV-Infected Individuals on ART

### 2.1. Persistent CVD Risk in HIV-Infected Individuals Despite ART

The HIV-associated risk of developing CVD persists despite the initiation of ART [3,4,5,6]. For example, atherosclerotic conditions continue to progress more in the HIV population after ART is initiated based on arterial carotid intima-media thickness [7]. Further, a more recent large meta-analysis combining five studies and 89,713 subjects concluded that “stroke represents a relatively common complication in young HIV patients” [8]. Another example is primary pulmonary arterial hypertension (PAH); the prevalence of PAH was determined as 0.5% during the pre-ART era [9] and stayed high (0.46%) in the era of ART [10]. These are alarming numbers, given that the prevalence of PAH in the general population is 0.0015%. An overview about studies assessing the risk of HIV patients on ART for developing CVD is depicted in Table 1.

### 2.2. Factors Contributing to CVD Risk in HIV-Infected Individuals on ART

What are the possible explanations for this persistently increased risk for CVD in the HIV-positive population? One possibility is toxicity and dyslipidemia from antiretroviral drugs, as reviewed previously [32]. Typically, nucleoside- and non-nucleoside-based reverse transcriptase inhibitors are combined, with or without the addition of integrase or HIV-protease-targeting inhibitors. The latter, in particular, have displayed pronounced toxicity and are now mostly out of use [33]. Typically, newer ART regimens have less toxicity and are more efficient than early HIV replication inhibitors [32,34].

Another possible explanation why inflammation persists during ART could be that HIV infection irreversibly damages the mesenteric lymphatic system. This way, microbial translocation may continue after ART initiation, possibly leading to chronic inflammation [35,36]. In this context, the link between systemic inflammation and vascular health was the topic of the “Strategic Timing of Anti-Retroviral Therapy” (START) trial, in which HIV patients on successful ART demonstrated a correlation between inflammatory plasma markers and impaired arterial elasticity [37]. Interestingly, elevated markers of endothelial dysfunction have been found in patients with ART-controlled viral loads [38].

Increased CVD risk in HIV-infected patients may also be caused by virus-induced dyslipidemia, which is highly prevalent among patients living with chronic HIV infection [39]. Interestingly, HIV-Nef was suggested as the main viral factor associated with HIV-linked dyslipidemia [40,41,42]. Because Nef protein production appears to be relatively resistant to ART, this viral protein could explain persistent dyslipidemia in ART patients (see upcoming chapters on HIV-Nef persistence).

Last but not least, HIV in latent or actively infected cells could elicit the production and release of EVs [43,44]. Studies have suggested EVs mediated cardiovascular dysfunction in transferring its protein and non-coding RNA cargo to vascular endothelial and smooth muscle cells [43,45]. Several reports have demonstrated that the HIV-encoded Nef protein organizes EV formation and uses them as vehicles for dissemination [46,47,48,49,50,51,52]. These Nef-containing EVs are toxic to surrounding cells, including bystander CD4 positive T cells and vascular endothelial cells that absorb them and become apoptotic [46,47,48,52]. These possible effects of HIV-associated EVs will be discussed in more detail in the following chapters.

## 3. Increased EV in HIV-Infected Individuals Despite ART

### 3.1. EVs Are Enriched in the Plasma of HIV Patients on ART

The term extracellular vesicles (EV) defines different types of vesicles secreted outside of the cell, including exosomes, microvesicles, and apoptotic bodies [53]. Because EVs are taken up by blood and tissue cells, they could spread proinflammatory cargo and HIV proteins throughout the body. Exosomes are reported as a subpopulation of EVs associated with HIV activities, including inflammation and dyslipidemia [54,55,56,57,58,59,60,61]. Of note, Lee et al. [62] showed that in HIV patients—whether viremic or on combined high dose antiretroviral therapy (ART)—EV numbers were approximately 20-fold higher over healthy controls. Similarly, the cargo load of these EVs from HIV increased 18–25-fold over otherwise healthy controls. As for cargo load, the authors determined the expression of miRNA and protein [62]. Support for the significance of these findings came from further studies using unbiased proteomic approaches and miRNA analysis to demonstrate that isolated plasma EVs contained markers “associated with immune activation and oxidative stress in HIV patients on ART” [44,63].

### 3.2. Nef Protein Persists in EVs from Body Fluids

Given the presence and upregulation of EVs in the HIV-positive population, “early response” HIV genes including Nef, tat, and rev were suspected. These “early” HIV genes decrease after initiation of ART treatment less prominently than other HIV gene products [64]. Intracellular mRNA-encoding Nef levels were maintained during ART, while Tat and Rev showed less prominent mRNA expression [65].

Nef protein inside CD45+ EVs in the plasma of HIV-infected individuals was first reported in 2011 by Raymond et al. [66]. Subsequent studies have detected Nef protein in EVs from plasma and peripheral blood mononuclear cells (PBMCs) of both treatment-naive HIV patients and HIV-infected individuals on ART with undetectable viral loads [47,67]. Interestingly, HIV-Nef was detected in over half the patients on ART and in elite controllers in a study using 134 HIV-infected patients with undetectable HIV RNA [68]. Our previous work could confirm these findings in a much smaller study of 16 patients on ART, in which also about half were HIV-Nef-positive [47]. Interestingly, we also found high numbers of Nef protein-positive PBMCs using flow cytometry and a panel of three monoclonal antibodies targeting three different and highly conserved epitopes because of the high mutation rate of HIV.

### 3.3. Nef Protein in EVs Transfers Rapidly to Blood and Tissue Cells

The presence of Nef in both EVs and PBMC begs the question of whether mononuclear cells are the target or source of Nef-containing EVs. Apparently, Nef can be transported by EVs and transferred to PBMCs and endothelial cells because it is present to a large extent in B cells and CD4− T cells, and Nef-positive PBMCs transferred Nef rapidly to vascular endothelial cells in vitro [47,67]. Further support for the hypothesis that mononuclear cells are the target of Nef-containing EVs comes from our previous work demonstrating the presence of Nef protein in EVs and cells derived from bronchoalveolar lavages (BAL) [46]. While 50% of the patients (10 out of 18) displayed Nef-EV positivity based on ELISA, Nef positive BAL cells were randomly distributed between alveolar macrophages, CD4+ T cells, and CD8+ T cells (in nine out of 15 HIV patients on ART).

To date, the source (or sources) of these surprisingly robust levels of Nef protein in successfully ART-treated patients remains unclear. Lee et al. [62] suggested a myeloid or myeloid-like compartment as the origin of Nef-induced EV formation, based on protein array analysis of patient-derived plasma EVs, which was different from those in EVs derived from HIV-transfected Jurkat cells. A closer identification of this compartment could help in understanding HIV latency in the era of ART.

## 4. HIV-Nef Induces Extracellular Vesicle-Assisted Inflammatory Load (EVAIL)

### 4.1. Specific Surface Proteins in EVs Define Proinflammatory Cargo Associated with HIV Proteins

Given the 20-fold increase in EV numbers and protein load found in plasma from HIV patients on ART, researchers asked whether HIV proteins could be linked to specific EV surface markers. To address this, Lee et al. [62,69] tested a panel of 262 monoclonal antibodies coupled to magnetic beads for their ability to adsorb plasma-derived EV with HIV cargo. They could identify one antibody (clone 2H4) that captured all EVs containing HIV proteins (Nef and Vpu). Interestingly, this antibody was identified to recognize activated αvβ3, an angiogenic integrin also known as vitronectin receptor, and associated with macrophages’ phagocytic activity. Further, this study found that activated αvβ3 positive EVs contained the proinflammatory matrix-metalloproteinases ADAM-17 (TACE) and ADAM-10, absent in the unbound fraction. Subsequently, Nef- and TACE-containing EVs were isolated from HIV patients, which, in turn, correlated with immune pathogenesis in chronic HIV-infected patients [62].

Interestingly, the presence of Extracellular Vesicle Assisted Inflammatory Load (EVAIL) includes a specific pattern consisting of cytokines, chemokines, and growth factors, such as PDGF BB and FGF-9 [62]. In conclusion, a particular EV surface protein emerged as a potential biomarker for HIV-associated EVs carrying inflammatory cargo. However, there is a gap of knowledge regarding the Nef-associated composition of non-coding RNAs, including miRNAs, which were reported to be modulated in HIV-induced EVs [43,44].

### 4.2. Nef Initiates Endosomal Routing Leading to Recruitment of ADAM-17/TACE and Secretion of TNF

Ostalecki et al. [70] showed that uptake of these Nef-containing EVs induced endosomal TNF cleavage by TACE. They identified as the first step Nef-dependent internalization of TACE in Rab4+ early endosomes. This enabled compartmentalization with transmembrane TNFα, which was followed by TACE activation, TNFα cleavage, and secretion as TNFα/TACE repackaged EVs [70]. TNFα maintains at intravesicular location, which is different from the canonical TNFα release as a soluble protein from cells. These findings are significant as TACE is the primary mechanism driving the release of mature TNFα and the primary driver of other proinflammatory pathways involved in chronic inflammation and its associated diseases [71]. They also show that Nef is associated with vesicle sorting. It is likely that Nef orchestrates the loading of proteases and cytokines in EVs. Alternatively, the host system could interpret Nef as a danger signal to load Nef-containing EVs with proteases and cytokines in preparation to fight infectious agents.

## 5. Pathophysiology of the HIV-Nef-Associated EVAIL

The elucidation of pathogenic mechanisms in HIV-associated comorbidities in the era of ART is still incompletely understood, as is the function of HIV-released EVs. The release of pathogenic HIV proteins like Nef into the systemic circulation through ADAM17/TACE-containing EVs could be advantageous for HIV by activating latent viral activity [72,73]. Likewise, ADAM-17/TACE-induced v generation can prime resting CD4+ T lymphocytes for HIV expression and replication [50,69,70]. As it would make evolutionary sense for the virus to promote its infectivity broadly, could it be that the same TACE-induced TNFα generation causes endothelial activation and dysfunction more like an unwanted side effect? Indeed, Nef-containing EVs can affect recipient cells both on functional and gene expression levels [46,47,48,62,74,75]. Khan et al. [76] isolated Nef-EVs from HIV patients with HIV-associated dementia (HAD) and demonstrated that they could elicit increased Aβ secretion from neuronal cells. Reportedly, Nef-containing EVs are indeed taken up by neuronal cells leading to oxidative stress [75].

EVs must not necessarily have to reach distal organs to increase proinflammatory pathologies. Alternatively, microglia cells could produce and release such EVs. Raymond et al. [77] demonstrated that EVs could cross the blood-brain barrier. In addition, they found that microglial cells transfected with Nef released Nef-EV to reduce the endothelial blood-brain barrier. This loss of barrier function is likely due to Nef-EV reducing tight junction protein expression, ZO-1. Importantly, this study demonstrated that by using a specific Nef-peptide inhibitor, Nef-EV-induced disturbance of their blood-brain barrier model can be reversed [77]. These findings indicate that HIV reservoir cells in the brain can employ HIV-Nef release to contribute to HIV-associated neurocognitive diseases (HAND). Furthermore, they could also promote cardiovascular disease as EVs produced from brain cells cross the BBB also in reverse.

As another example of the proinflammatory propensity of EVs, the addition of Nef-EVs to peripheral blood monocytes (PBMCs) led to TNFα-converting enzyme (TACE/ADAM17) packaging into vesicles and subsequent secretion [69]. Uptake of Nef-EVs in T cells from human PBMCs causes free radical formation and apoptosis when these cells are brought in close contact with human coronary arterial endothelial cells. This finding suggests that leukocytes can further enhance HIV-Nef-induced pathologies [47].

Wang et al. [48] were the first to report that Nef is transported from T cells to endothelial cells to cause reactive oxygen species (ROS) production, upregulation of chemokines, and pro-apoptotic signaling. Interestingly, T cells infected with HIV elicited the same panel of endothelial activation markers, which was either absent or much reduced when HIV with a deleted Nef gene was used.

Further, Nef-containing EVs from BAL fluids and Nef-transfected cells were shown to induce programmed cell death in endothelial cells, dependent on induction of EMAP II [46]. Interestingly, transgenic expression of Nef in the endothelium induced endothelial EMAP II surface expression, which could mediate the emphysema phenotype in these transgenic mice.

Furthermore, our own previous work has shown that HIV-Nef expressing T cells and HIV-Nef-induced EVs upregulated endothelial adhesion proteins and apoptosis. This upregulation occurred together with cytosolic dyes and Nef protein transfer from T to endothelial cells, dependent on Rac1-activation [47]. These Rac1-dependent activities were characterized by the production of NADPH/NOX2-mediated reactive oxygen species (ROS). Statin treatment equally inhibited Rac1 inhibition in preventing or reversing HIV-Nef-induced ROS formation, mitochondrial polarization, and increased pro-apoptotic signaling in human coronary arterial endothelial cells [47]. This therapeutic effect was explained by the ability of statins to block Rac1 prenylation. Indeed, geranylgeranyl transferase inhibitors significantly reduced HIV-Nef-induced ROS formation. These findings suggest a protective role of statins beyond lipidemia. Certainly, there is clinical evidence of the positive effects of statins in combination with ART in HIV/AIDS patients, thus the recommendation that statin treatment should be considered in this population after due attention to possible drug–drug interactions [78].

In addition to the ability of Nef-EVs to induce endothelial cell activation and increased stickiness of proinflammatory leukocytes to vascular endothelial cells even under conditions of flow [47], they can increase cholesterol and triglyceride levels [40,41,42]. Cholesterol levels are known to be regulated by reverse cholesterol transport. Reportedly, Nef mediates the down-regulation of adenosine triphosphate-binding cassette transporter A1 (ABCA1) transporters by post-translational degradation [42]. In apoE (−/−) mice, injected Nef reduced levels of ABCA1 in the liver, supporting the link between Nef and increased cholesterol plasma levels [41]. Not surprisingly, these effects occurred together with increased atherosclerotic lesion size and blood vessel remodeling.

Furthermore, the results of Nef on dyslipidemia could be replicated in the SIV-infected macaque model [40]. SIV infection down-regulated the ABCA1 cholesterol transporter in the liver, and serum from SIV-infected but not from healthy macaques inhibited cholesterol efflux from human cultivated macrophages. Interestingly, Nef-induced ABCA1 downregulation goes beyond regulation of lipid levels: Mukhamedova et al. [79] could demonstrate that Nef-induced ABCA1 downregulation results in increased abundance of lipid rafts in monocytes in a Cdc42 dependent fashion. The authors also found that these changes in lipid rafts stimulate inflammation through “re-localization of TLR4 and TREM-1 to rafts”.

In summary, Nef-containing EVs can contribute to CVD both by increasing proteolytic and proinflammatory cargo and by intrinsic functions of the Nef protein (see also Figure 1). It would be interesting to study other viruses for a similar mechanism to employ EVs to control immune-related functions.

## 6. Does the EVAIL Concept also Hold True for Other Viruses? What about Pandemic SARS CoV-2?

It would be interesting to test whether mechanisms similar to HIV-Nef occur in other virus-related diseases. The COVID-19 respiratory illness is associated with pulmonary vascular thickening, endothelial dysfunction, thrombo-inflammation, and micro-thrombi [80,81]. Emerging findings suggest that COVID-19 patients, like HIV-infected patients, may be at higher risk of developing pulmonary hypertension in the future [82]. When A549 lung epithelial cells were transduced with lentivirus encoding SARS-CoV-2 proteins, exosomes are released carrying these viral proteins. The addition of these exosomes to recipient pluripotent stem cell-derived cardiomyocytes resulted in the entry of viral RNA into cardiomyocytes and increased expression of inflammatory genes [83]. It remains unclear whether these passed viral genes are biologically active and capable of inducing cell injury. It may be too speculative, but cellular recipients of EVs carrying viral proteins could be prone to becoming targets of autoimmune reactions.

Exosomes also have been reported to transfer ACE2, the receptor exploited by SARS-CoV-2 for cell entry, to recipient cells [84]. Interestingly, a higher burden of EV-associated ACE2 is noted in SARS-CoV-2-infected patients without hypoxia than those requiring oxygen treatment. This finding suggests that EVs with ACE2 cargo might act as decoys, thereby reducing SARS-CoV-2 infection in epithelial cells [85,86]. Alternatively, EVs transporting ACE2 cargo may also render ACE2-negative cells, usually not targeted by SARS-CoV-2, to become infected with this virus. Although there is no apparent means of interfering with ACE2 transport by EVs, this is an essential finding that, depending on the membrane orientation of this receptor, could lead to antibody-directed detection and potential removal of COVID-19-related EVs.

It has been shown (see previous sections on HIV) that HIV-associated EVs carry HIV proteins and proinflammatory cargo that can be transferred to endothelial and other tissue recipient cells. Therefore, it would be interesting to explore whether this EV-mediated cargo transfer can also be observed with SARS-CoV-2-associated EVs. Our recent work has shown that EVs from patients infected with SARS-CoV-2 display higher levels of specific cytokines in correlation with disease severity. For example, large EVs from the plasma of patients with COVID-19 are enriched with multiple members of the TNF superfamily and their receptors and IL-6 family proteins [86]. These cytokines and their receptors are also characteristic of acquired respiratory distress syndrome (ARDS). Specifically, IL-6 has been associated with cytokine release in ARDS, septic shock, and COVID-19 [87,88,89]. Notably, the transfer of cytokine receptors could explain why EVs may add to inflammation despite the presence of already high concentrations of cytokines in COVID-19. This would imply that these cytokines receptors would be functionally integrated into the lipid bilayers of EVs. Of note, this is in contrast to soluble cytokine receptors in plasma, which typically result from proteolytic cleavage of the extracellular domains. Although uptake of these receptors has not yet been addressed in the study of COVID-19-associated EVs, it is a tempting speculation that COVID-19-related EVs could heighten the cytokine storm by increasing proinflammatory receptor densities in blood and other vascular cells.

The finding of increased IL-6 in COVID-19-associated EVs is significant because increased IL-6 plasma levels are part of predictor panels for cardiac events, especially in the setting of HIV infection [90,91,92]. In addition, higher levels of pro-inflammatory RAGE and EN-RAGE in EVs correlated with COVID-19 clinical severity could further provide a positive feedback loop for generating a cytokine storm [86]. Additionally, several proteins and proteases associated with cardiovascular disease and vascular remodeling, including prostatin, cathepsin L1, matrix metalloproteinase 9, and carboxypeptidases 1/2 (CPA1/CPB1), are also upregulated in large EVs from patients with severe infection [86].

EVs, transiently released from injured cardiomyocytes, contain markers of early ischemic injury [93,94,95]. This release may comprise a mechanism by which some patients with COVID-19 experience elevated levels of circulating cardiac troponins (cTn). Lala et al. reported a significantly higher mortality risk among COVID-19 patients with even a tiny troponin leak [96]. EV-linked tissue factor (TF), a vital driver of the extrinsic coagulation cascade, is also elevated in COVID-19 patients with moderate [97] and severe disease [86]. Elevated TF in EV levels have been already linked to cancer-associated thrombosis [98] and now our work correlated EV-linked TF levels and activity with increasing COVID-19 disease severity and length of hospitalization [86]. This suggests that the prothrombotic and antithrombotic molecular profile of EVs from SARS-CoV-2-infected individuals may be one of the factors responsible for the increased risk of microthrombosis COVID-19 infection (see Figure 1). The long-term cardiovascular consequences of SARS-CoV-2 infection may result from an EV-mediated prothrombotic environment and endothelial activation and dysfunction. Notably, EVs may contribute to COVID-19 disease severity by adding increased proteolytic stress and procoagulant activation pathways to the already known increases in inflammatory cytokines. Additional research is needed to explore whether there is a role for EVs in propagating tissue destruction, inflammation, and thrombosis weeks to months out from COVID-19 diagnosis. Finally, it needs to be established whether EV cargo could become biomarkers for disease severity in COVID-19 or if distinct surface markers for COVID-19-associated EVs could be targeted for their therapeutic removal.

## 7. Conclusions and Potential for Therapy

This review focuses on a single protein, Nef, as an example of how viruses and their proteins can trigger the specific release of proinflammatory EVs. Although further studies targeting these EVs are required to provide more direct links to CVD, the potential of EVAIL to activate the endothelium is intriguing. It may be feasible to target the generation of EVAIL if we better understand the generation and uptake of EVs. Interestingly, EV-associated anti-inflammatory load has been described from mesenchymal stem cells (MSCs) [99,100]. The anti-inflammatory activities of MSC have emerged as an attractive anti-inflammatory strategy over the past decade [101,102,103]. In this article, MSCs were suggested to improve cardiovascular function and reduce the exuberant production of influenza-induced cytokines [102]. MSCs are already in clinical trials for treating COVID-19, and using EVs from these cells could be added to other already used MSC-based therapeutics. In addition, the incorporation of other anti-inflammatory and immune regulatory proteins like CD24 or cytokine-specific neutralizing therapeutic antibodies could lead to a novel platform of molecular therapies mediated by EVs.

## Figures and Tables

**Table 1:** List of studies describing the increased risk for various CVD in HIV patient cohorts on Anti-Retroviral Therapy.

Study	Country	No. of HIV + Patients	Outcome
Lang et al. [11] (2010)	France	74,958	Myocardial Infarction (Standardized Morbidity Ratio 1.5)
Hsue et al. [12] (2010)	USA	196	Left Ventricular Mass Index (77.2 g/m (2) vs. 66.5 g/m (2) *p* < 0.0001)
			Diastolic Dysfunction (50% vs. 29% *p* = 0.008)
Durand et al. [13] (2010)	Canada	7053	Acute Myocardial Infarction (Adjusted Incidence Ratio 2.11)
Butt et al. [14] (2011)	USA	2391	Heart Failure (Hazard Ratio 1.81)
Sliwa et al. [15] (2012)	South Africa	518	Pericarditis (13% vs. 1.2%, *p* < 0.001)
Lorgis et al. [16] (2012)	USA	608	Ischemic Cardiomyopathy (7.6% vs. 4.2% *p* = 0.02)
Freiberg et al. [17] (2013)	USA	27,350	Acute Myocardial Infarction (Hazard Ratio 1.48)
Womack et al. [18] (2014)	USA	710	Cardiovascular Disease Outcome (Hazard Ratio = 2.8)
Luo et al. [19] (2014)	China	325	Diastolic Dysfunction (Prevalence 16.5% vs. 7.2%, *p* = 0.027)
Fontes-Carvalho et al. [20] (2015)	Portugal	206	Diastolic Dysfunction (Prevalence 19% vs. 3.3%, *p* < 0.05)
Rasmussen et al. [21] (2015)	Denmark	3251	Myocardial Infarction (Incidence Rate Ratio 1.78)
Chow et al. [22] (2015)	USA	100	Subclinical Atherosclerosis (mean Coronary Artery Calcium Score, Relative Risk = 1.20, *p* < 0.05)
Al-Kindi et al. [23] (2016)	USA	36,400	Heart Failure (Relative Risk 1.66)
Freiberg et al. [24] (2017)	USA	31,523	HFpEF (Hazard Ratio 1.21)
			HFrEF (Hazard Ratio 1.61)
Knudsen et al. [25] (2018)	Denmark	908	Peripheral Artery Disease (Adjusted Odds Ratio 1.9)
Alonso et al. [26] (2019)	USA	19,798	Stroke (Hazard Ratio 2.3)
			Myocardial Infarction (Hazard Ratio 1.2)
			Heart Failure (Hazard Ratio 2.8)
			Atrial Fibrillation (Hazard Ratio1.3)
Beckman et al. [27] (2019)	USA	28,714	Peripheral Artery Disease (Hazard Ratio 1.19)
Rao et al. [28] (2019)	Various	248,145 *	Acute Myocardial Infarction (Relative Risk 1.96)
Chattranukulchai et al. [29]	Thailand	298	Diastolic Dysfunction (Adjusted Odds Ratio 3.5)
			LV Hypertrophy (Adjusted Odds Ratio 1.91)
Gaspar Vallilo et al. [30]	Brazil	148	LV Dilation and ART (Adjusted Odds Ratio 0.98)
			Septal Hypertrophy and ART (Adjusted Odds Ratio 0.96)
Shen et al. [31]	China	587	ST-T segment elevation and ART (Adjusted Odds Ratio 0.96)
			All ECG abnormality and ART (Adjusted Odds Ratio 0.95)

* = pooled meta-analysis from 16 studies.

## Data Availability

Not applicable.
